# Differential Features of Culprit Intracranial Atherosclerotic Lesions: A Whole‐Brain Vessel Wall Imaging Study in Patients With Acute Ischemic Stroke

**DOI:** 10.1161/JAHA.118.009705

**Published:** 2018-07-22

**Authors:** Fang Wu, Qingfeng Ma, Haiqing Song, Xiuhai Guo, Marcio A. Diniz, Shlee S. Song, Nestor R. Gonzalez, Xiaoming Bi, Xunming Ji, Debiao Li, Qi Yang, Zhaoyang Fan, the WISP Investigators

**Affiliations:** ^1^ Department of Radiology Xuanwu Hospital Capital Medical University Beijing China; ^2^ Department of Neurology Xuanwu Hospital Capital Medical University Beijing China; ^3^ Department of Neurosurgery Xuanwu Hospital Capital Medical University Beijing China; ^4^ Biostatistics and Bioinformatics Research Center Cedars‐Sinai Medical Center Los Angeles CA; ^5^ Department of Neurology Cedars‐Sinai Medical Center Los Angeles CA; ^6^ Department of Neurosurgery Cedars‐Sinai Medical Center Los Angeles CA; ^7^ Siemens Healthineers Los Angeles CA; ^8^ Biomedical Imaging Research Institute Cedars‐Sinai Medical Center Los Angeles CA; ^9^ Departments of Medicine and Bioengineering University of California Los Angeles CA

**Keywords:** high‐resolution magnetic resonance imaging, intracranial atherosclerosis, stroke, vessel wall imaging, Atherosclerosis, Cerebrovascular Disease/Stroke, Magnetic Resonance Imaging (MRI)

## Abstract

**Background:**

Intracranial atherosclerotic disease tends to affect multiple arterial segments. Using whole‐brain vessel wall imaging, we sought to study the differences in plaque features among various types of plaques in patients with a recent unilateral anterior circulation ischemic stroke.

**Methods and Results:**

Sixty‐one patients with unilateral anterior circulation ischemic stroke were referred to undergo whole‐brain vessel wall imaging (before and after contrast) within 1 month of symptom onset for intracranial atherosclerotic disease evaluations. Each plaque was classified as a culprit, probably culprit, or nonculprit lesion, according to its likelihood of causing the stroke. The associations between plaque features (thickening pattern, plaque‐wall contrast ratio, high signal on T1‐weighted images, plaque contrast enhancement ratio, enhancement grade, and enhancement pattern) and culprit lesions were estimated using mixed multivariable logistic regression after adjustment for maximum wall thickness. In 52 patients without motion corruption in whole‐brain vessel wall imaging, a total of 178 intracranial plaques in the anterior circulation were identified, including 52 culprit lesions (29.2%), 51 probably culprit lesions (28.7%), and 75 nonculprit lesions (42.1%). High signal on T1‐weighted images (adjusted odds ratio, 9.1; 95% confidence interval, 1.9–44.1; *P*=0.006), grade 2 (enhancement ratio of plaque ≥ enhancement ratio of pituitary) contrast enhancement (adjusted odds ratio, 17.4; 95% confidence interval, 1.8–164.9; *P*=0.013), and type 2 (≥50% cross‐sectional wall involvement) enhancement pattern (adjusted odds ratio, 10.1; 95% confidence interval, 1.3–82.2; *P*=0.030) were independently associated with culprit lesions.

**Conclusions:**

High signal on T1‐weighted images, grade 2 contrast enhancement, and type 2 enhancement pattern are associated with cerebrovascular ischemic events, which may provide valuable insights into risk stratification.

**Clinical Perspective**

**What Is New?**

- A recently developed whole‐brain 3‐dimensional vessel wall imaging technique was used in our study because of its improved spatial coverage, T1 contrast weighting, and signal suppression of cerebrospinal fluid.
- We identified the significant differences in plaque features among culprit/probably culprit/nonculprit lesions.

**What Are the Clinical Implications?**

- Our results provide strong evidence on further research into the potential of these features as important imaging markers for better risk stratification and decision making for early intervention.

## Introduction

Intracranial atherosclerotic disease (ICAD) is one of the most common causes of stroke worldwide, with particularly high prevalence in Asians, Hispanics, and Africans.^1^, ^2^, ^3^, ^4^, ^5^ ICAD tends to affect multiple arterial segments.^6^ Despite the same genetic milieu and acquired factors, the coexisting atherosclerotic plaques in a given patient exhibit different outcomes; some lesions become symptomatic, whereas others remain asymptomatic.^7^ This fact brings 2 critical challenges in the management of patients with ICAD: the identification of the symptomatic or culprit lesion in symptomatic patients and the identification of the vulnerable (or high risk for causing a stroke) plaque in asymptomatic patients. The solution currently available to clinicians heavily relies on luminography imaging, with which the stenotic degree is a primary criterion for defining a culprit or high‐risk lesion. However, extensive research on the atherosclerosis disease has suggested that lumen narrowing may not be a reliable indicator of plaque severity because of positive remodeling of the vessel wall,^8^ and the features exhibited directly by plaques may provide complementary information.^9^, ^10^

Intracranial magnetic resonance (MR) vessel wall imaging (VWI) has emerged as a noninvasive modality for directly characterizing ICAD.^10^ The advent of 3‐dimensional VWI techniques in the past few years also makes it possible to simultaneously assess atherosclerotic plaques presenting in multiple arterial segments.^11^, ^12^ Using VWI, several plaque features have been intimately associated with symptomatic or asymptomatic lesions, such as high signal on T1‐weighted images (HST1), wall thickening pattern, and postcontrast enhancement.^7^, ^13^, ^14^, ^15^, ^16^, ^17^, ^18^ However, most of previous studies were limited to one feature only, a local arterial segment, or a cohort with heterogeneous time periods after symptom onset. Targeting a homogeneously acute stroke patient cohort and understanding the differences in a group of plaque features between symptomatic and asymptomatic lesions may yield an enhanced ability to identify the culprit or high‐risk lesion, thus providing more clinically relevant insights into risk stratification and treatment decision.^19^

The present study sought to use a recently developed whole‐brain 3‐dimensional VWI technique (WB‐VWI) to assess a cohort of patients with a recent unilateral anterior circulation ischemic stroke. The technique, based on sampling perfection with application‐optimized contrast using different flip angle evolutions, coupled with nonselective excitation and a trailing magnetization flip‐down module, has demonstrated improved spatial coverage, T1 contrast weighting, and signal suppression of cerebrospinal fluid. In principle, these all favor the delineation of the aforementioned plaque features.^20^, ^21^ The atherosclerotic lesions in all large intracranial arteries involved in bilateral anterior circulation were investigated to reveal the plaque features that are highly associated with symptomatic lesions.

## Methods

The data that support the findings of this study are available from the corresponding author on reasonable request.

### Patients

The study was approved by the Ethics Committee of Xuanwu Hospital of Capital Medical University (Beijing, China), and informed consent was obtained from all patients or patient guardians. From October 2015 to June 2017, patients who were admitted to the stroke center of our hospital were prospectively recruited into this study if they had the following: (1) unilateral ischemic stroke in the territory of the anterior circulation within the past 30 days; (2) at least one ≥50% intracranial stenosis within the vascular territory of the stroke, as confirmed by MR angiography, computed tomography angiography, or digital subtraction angiography; or (3) at least one atherosclerotic risk factor, including hypertension, diabetes mellitus, hyperlipidemia, and cigarette smoking. Exclusion criteria were as follows: (1) contraindications to MR and gadolinium‐based contrast agents; (2) history of strokes or transient ischemic attacks in the contralateral anterior circulation territory; (3) coexistent ≥50% ipsilateral extracranial carotid artery stenosis; (4) nonatherosclerosis vasculopathy (eg, dissection, vasculitis, or Moyamoya disease); (5) evidence of cardioembolism; and (6) history of transluminal intervention.

Clinical information, including sex, age, body mass index, systolic blood pressure, diastolic blood pressure, total cholesterol, low‐density lipoprotein, high‐density lipoprotein, current smoking, and onset to WB‐VWI time, was recorded for each patient.

### Imaging Protocol

Imaging was performed on a 3‐T system (Magnetom Verio; Siemens, Erlangen, Germany) equipped with a standard 32‐channel head coil. The protocol consisted of 3‐dimensional time‐of‐flight MR angiography and precontrast and postcontrast 3‐dimensional T1‐weighted WB‐VWI. Imaging parameters for WB‐VWI were as follows: repetition time/echo time, 900/15 ms; field of view, 170×170 mm^2^; 240 slices; voxel size, 0.53×0.53×0.53 mm^3^ without any interpolation; and scan time, 8 minutes. Postcontrast WB‐VWI was performed 5 minutes after the injection of single‐dose (0.1 mmol/kg of body weight) gadolinium‐based contrast agent (Magnevist; Schering, Berlin, Germany).

### Image Analysis

Commercial software (Osirix MD; Pixmeo SARL, Bernex, Switzerland) with 3‐dimensional multiplanar reformation was used for image analysis. Source images and reformatted longitudinal and cross‐sectional views were all available to readers.

WB‐VWI images were first evaluated by an experienced neuroradiologist (F.W.) who was blinded to the identity and clinical information of patients. All large intracranial internal arteries of bilateral anterior circulation, including the intracranial internal carotid artery, middle cerebral artery (up to the M3 segment), and anterior cerebral artery, were inspected for the presence of atherosclerotic plaques by longitudinal and cross‐sectional views. A plaque was defined as wall thickening on the cross‐sectional view of a segment using its adjacent proximal, distal, or contralateral vessel segment as a reference. The thickening pattern was visually categorized as type 1 (<50% cross‐sectional wall involvement) or type 2 (≥50% cross‐sectional wall involvement).

Quantitative and qualitative evaluations were performed on the cross‐sectional views of individual lesions. Mean signal intensity (SI) was quantified with manually drawn regions of interest. The mean SIs of the brightest region within each identified atherosclerotic lesion and the SI of the reference normal vessel wall were measured on precontrast images. The ratio of these 2 measurements, named plaque‐wall contrast ratio (CR), was calculated. A plaque with the HST1 feature was identified if its plaque‐wall CR >150%. In addition, mean SI was quantified in each of plaques at their most thickened cross sections and also in their adjacent reference normal vessel wall, brain parenchyma, and the pituitary infundibulum on both precontrast and postcontrast images. Contrast enhancement ratio (ER), calculated as SI_postcontrast_/SI_precontrast_×100%, was determined for the plaque, reference wall, and pituitary infundibulum, where SI_precontrast_ and SI_postcontrast_ were the specific tissue's SI normalized by the SI of adjacent brain parenchyma on precontrast and postcontrast images, respectively.^15^ The degree of lesion enhancement was graded as follows: grade 0, plaque ER ≤ reference wall ER; grade 1, reference wall ER < plaque ER < pituitary infundibulum ER; grade 2, plaque ER ≥ pituitary infundibulum ER. The enhancement pattern was also visually categorized as type 1 (<50% cross‐sectional wall involvement) or type 2 (≥50% cross‐sectional wall involvement). In addition, maximum wall thickness was measured manually at the most thickened location of each plaque on the cross‐sectional precontrast images. The stenosis degree was defined as follow: 1−(lesion lumen area/reference lumen area)×100%. The stenosis degree was visually graded: 1, <25%; 2, 25% to 49%; 3, 50% to 74%; and 4, ≥75%.

A subgroup of patients (n=20) were included to study the reproducibility of the above quantitative measurements. A second observer performed quantification for assessing interobserver agreement, and observer 1 requantified the measures 8 weeks after the first reading session for assessing intraobserver agreement.

All detected atherosclerotic lesions were classified into 3 groups (culprit, probably culprit, and nonculprit), according to their likelihood of causing the stroke by 2 independent neurologists (Q.M., H.S.). They were provided each patient's clinical history, routine brain MR, angiographic images, and plaque locations, as determined by neuroradiologists (F.W., Q.Y.), but had no access to VWI data to avoid influence of their decision by postcontrast wall enhancement. A culprit lesion was defined as the following: (1) the only lesion within the vascular territory of the stroke or (2) the most stenotic lesion when multiple plaques were present within the same vascular territory of the stroke.^15^ A lesion was considered probably a culprit plaque if it was within the same vascular territory of the stroke but was not the most stenotic lesion.^15^ A lesion was deemed a nonculprit plaque when it was not within the vascular territory of the stroke.^15^ Any disagreements between the 2 reviewers were resolved with discussion and consensus.

### Statistical Analysis

All continuous variables were expressed as means±SDs, and categorical variables were summarized as counts (percentages). The differences in stenosis degree and plaque features (thickening pattern, plaque‐wall CR, HST1, plaque ER, enhancement grade, and enhancement pattern) among culprit, probably culprit, and nonculprit lesions were compared using a likelihood test between logistic models, with pairwise comparisons corrected using Holm correction. Odds ratios (ORs) of culprit (versus probably culprit or nonculprit lesions) with their respective 95% confidence intervals (CIs) were assessed using mixed multivariable logistic regression, with plaque features as covariates, and adjusted by maximum wall thickness. Random effects to describe multiple lesions in the same patient were used. Intraobserver and interobserver agreements in quantitative measurements were assessed by intraclass correlation coefficients, with a 1‐way random effect for intraobserver continuous variables and a 2‐way random effect for interobserver continuous variables. *P*<0.05 indicated statistical significance. All statistical analyses were performed by using SPSS, version 22.0 (IBM, Armonk, NY) and R, version 3.4.1.

## Results

### Patient Characteristics

Among the 61 patients who were recruited and scanned, 9 were excluded because of poor‐quality WB‐VWI images, caused by motion artifacts during either precontrast or postcontrast scans. A total of 52 patients (35 men and 17 women; mean age, 49.4±11.6 years) were finally included for analyses. The median interval between symptom onset and WB‐VWI was 11.9±7.3 days (range, 1–30 days). All patients had positive findings on diffusion‐weighted imaging sequences. Patient demographics and main atherosclerosis risk factors are summarized in Table 1.

**Table 1:** Patient Characteristics

Characteristics	Values
No. of patients^a^	52
Age, y	49.4±11.6
Male sex^a^	35 (67.3)
Body mass index, kg/m^2^	26.1±3.4
Systolic blood pressure, mm Hg	143.2±17.2
Diastolic blood pressure, mm Hg	84.5±10.7
Total cholesterol, mmol/L	3.8±1.2
LDL cholesterol, mmol/L	2.0±0.9
HDL cholesterol, mmol/L	1.1±0.3
Current smoker^a^	25 (48.1)
Onset to WB‐VWI time, d	11.9±7.3 (range 1–30)

Except where indicated, data are mean±SD. HDL indicates high‐density lipoprotein; LDL, low‐density lipoprotein; WB‐VWI, whole‐brain vessel wall imaging.

a Data are the number (percentage) of patients.

### Plaque Location and Classification

A total of 178 intracranial atherosclerotic lesions were identified in the 52 patients, with multiple lesions seen in 48 (92.3%). Of these lesions, 27 (15.2%) were found in the intracranial carotid artery, 148 (83.1%) were found in the middle cerebral artery, and 3 (1.7%) were found in the anterior cerebral artery (Table 2). A total of 107 lesions (60.1%) were located on the symptomatic side, and 71 (39.9%) were located on the asymptomatic side. A total of 52 lesions (29.2%) were deemed culprit lesions, 51 (28.7%) were deemed probably culprit lesions, and 75 (42.1%) were deemed nonculprit lesions.

**Table 2:** Plaque Distribution

Location	Symptomatic Side	Asymptomatic Side	Total
Intracranial ICA	15 (14.0)	12 (16.9)	27 (15.2)
MCA			
M1 segment	52 (48.6)	36 (50.7)	88 (49.4)
M2 segment	35 (32.7)	20 (28.2)	55 (30.9)
M3 segment	4 (3.7)	1 (1.4)	5 (2.8)
ACA			
A1 segment	1 (0.9)	2 (2.8)	3 (1.7)
Total	107	71	178

Data are given as number (percentage) of plaques. ACA indicates anterior cerebral artery; ICA, internal carotid artery; MCA, middle cerebral artery.

### Plaque Features in the 3 Lesion Types

The details of plaque features in the 3 lesion types are described in Table 3. The incidence of type 2 thickening pattern was significantly higher (*P*<0.001 for both) in culprit lesions (76.9%) than in probably culprit lesions (29.4%) and nonculprit lesions (18.7%). The wall thickening pattern showed no difference between probably culprit lesions and nonculprit lesions (*P*=0.162).

**Table 3:** Comparison of Vessel Wall Features Among 3 Types of Plaques

Variable	Culprit Lesions	Probably Culprit Lesions	Nonculprit Lesions	*P* Value			
					Culprit Lesions vs Probably Culprit Lesions	Culprit Lesions vs Nonculprit Lesions	Probably Culprit Lesions vs Nonculprit Lesions
Lesion	52 (29.2)	51 (28.7)	75 (42.1)				
Stenosis degree, %				<0.001	<0.001	<0.001	0.066
<25	0 (0)	30 (58.8)	59 (78.7)				
25–49	0 (0)	9 (17.6)	10 (13.3)				
50–74	21 (40.4)	8 (15.7)	4 (5.3)				
≥75	31 (59.6)	4 (7.8)	2 (2.7)				
Thickening pattern				<0.001	<0.001	<0.001	0.162
Type 1	12 (23.1)	36 (70.6)	61 (81.3)				
Type 2	40 (76.9)	15 (29.4)	14 (18.7)				
HST1	39 (75.0)	12 (23.5)	25 (33.3)	<0.001	<0.001	<0.001	0.232
Plaque‐wall CR^a^	2.0±1.0	1.4±0.4	1.4±0.4	<0.001	<0.001	<0.001	0.712
Enhancement degree				<0.001	0.001	<0.001	0.038
Grade 0	3 (5.8)	12 (23.5)	21 (28.0)				
Grade 1	23 (44.2)	30 (58.8)	51 (68.0)				
Grade 2	26 (50.0)	9 (17.7)	3 (4.0)				
ER_plaque_ a	1.8±0.6	1.4±0.5	1.2±0.3	<0.001	<0.001	<0.001	0.006
Enhancement pattern				<0.001	<0.001	<0.001	0.096
Type 1	14 (26.9)	40 (78.4)	67 (89.3)				
Type 2	38 (73.1)	11 (21.6)	8 (10.7)				

Except where indicated, data are the number (percentage) of lesions. All *P* values were estimated using a likelihood test between logistic models, with pairwise comparisons corrected using Holm correction. CR indicates contrast ratio; ER_plaque_, plaque enhancement ratio; HST1, the feature characterized as high signal on T1‐weighted images.

a Data are mean±SD.

**Figure 1:**
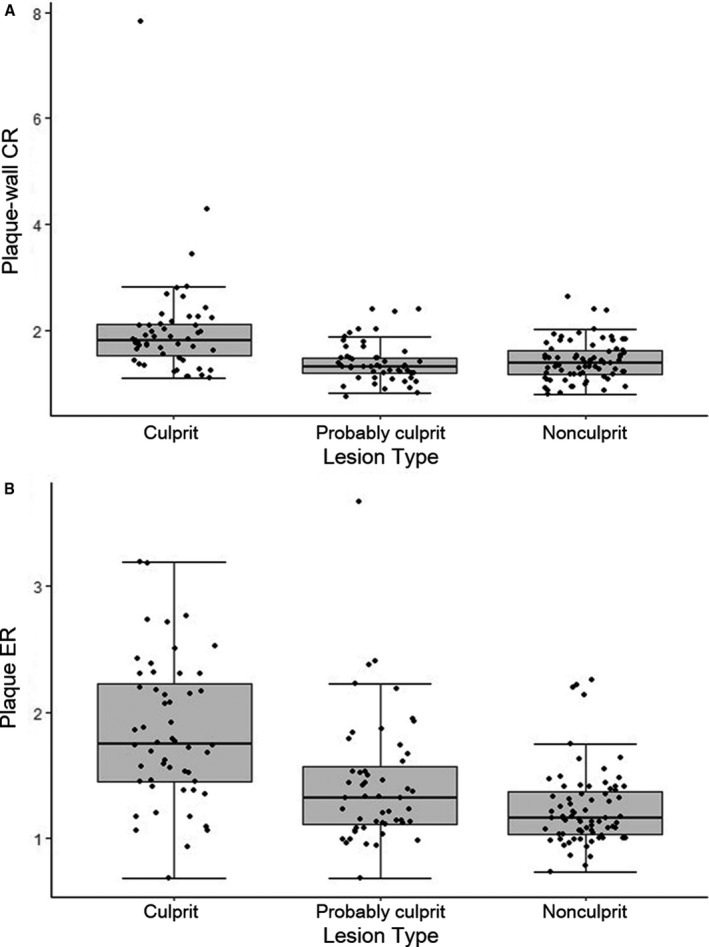
A, Comparison of plaque‐wall contrast ratio (CR) among 3 types of plaque. B, Comparison of plaque enhancement ratio (ER) among 3 types of plaque.

**Figure 2:**
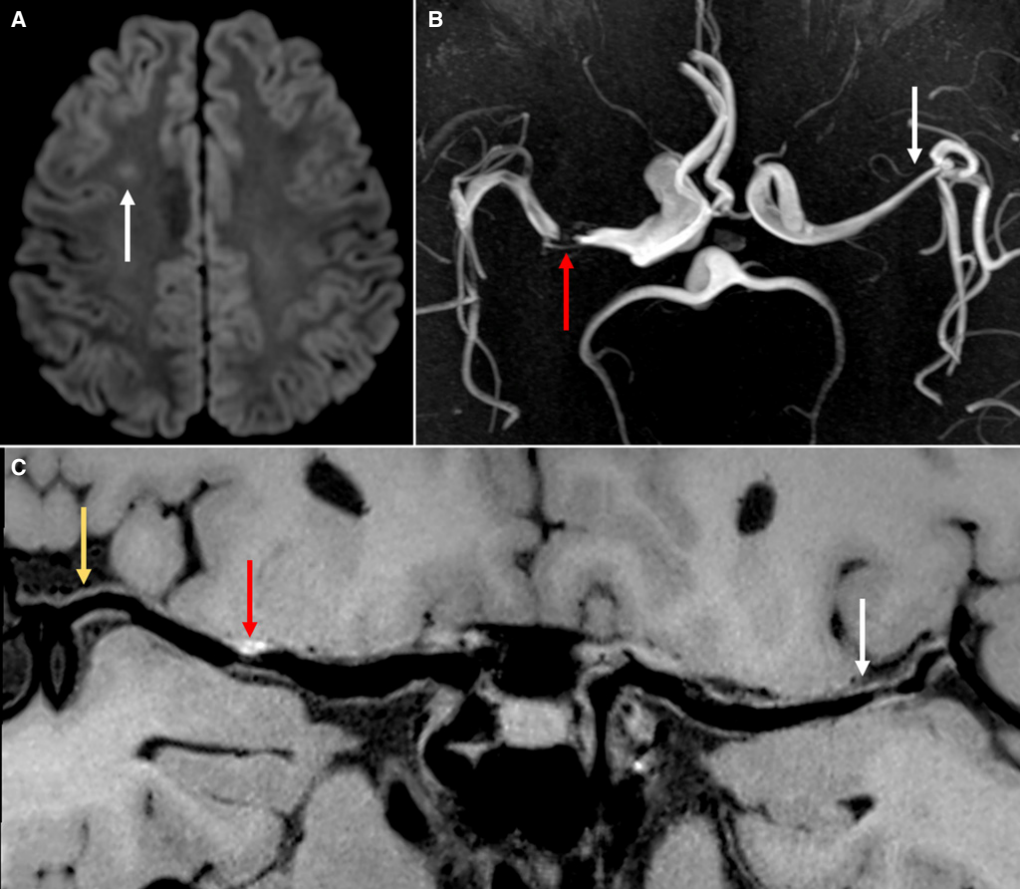
A 46‐year‐old male patient presented with right ischemic stroke. A, Axial diffusion‐weighted imaging detects a slightly high signal intensity lesion (white arrow) in the right centrum ovale. B, Time‐of‐flight magnetic resonance angiography shows a severe stenosis (red arrow) in the right middle cerebral artery (MCA) and a mild stenosis (white arrow) in the left MCA. C, Curved planar reconstruction image, based on the precontrast whole‐brain vessel wall imaging, shows a plaque with HST1 (red arrow) in the culprit plaque of right MCA‐M1. Plaques without high signal on T1‐weighted images are detected in the probably culprit plaque of the right MCA M2 segment (gold arrow) and the nonculprit plaque of the left MCA M1 segment (white arrow).

HST1 occurred in 75.0% of culprit lesions, 23.5% of probably culprit lesions, and 33.3% of nonculprit lesions. Compared with probably culprit lesions (*P*<0.001) and nonculprit lesions (*P*<0.001), culprit lesions were more presented with HSTI. The incidence of HST1 was not different between probably culprit lesions and nonculprit lesions (*P*=0.232). Plaque‐wall CR was significantly higher (*P*<0.001 for both) in culprit lesions (2.0±1.0) than in probably culprit lesions (1.4±0.4) and nonculprit lesions (1.4±0.4), and it was similar between probably culprit and nonculprit lesions (*P*=0.712) (Figure 1A). A representative case is shown in Figure 2.

The degree of contrast enhancement of culprit lesions (44.2%, grade 1; and 50.0%, grade 2) was significantly different from that of probably culprit lesions (58.8%, grade 1; and 17.7%, grade 2; *P*=0.001) and nonculprit lesions (68.0%, grade 1; and 4.0%, grade 2; *P*<0.001). The enhancement degree was also different between probably culprit lesions and nonculprit lesions (*P*=0.038). Plaque ER was significantly higher (*P*<0.001 for both) in culprit lesions (1.8±0.6) than in probably culprit lesions (1.4±0.5) and nonculprit lesions (1.2±0.3), and it was also significantly different between probably culprit and nonculprit lesions (*P*=0.006) (Figure 1B).

**Figure 3:**
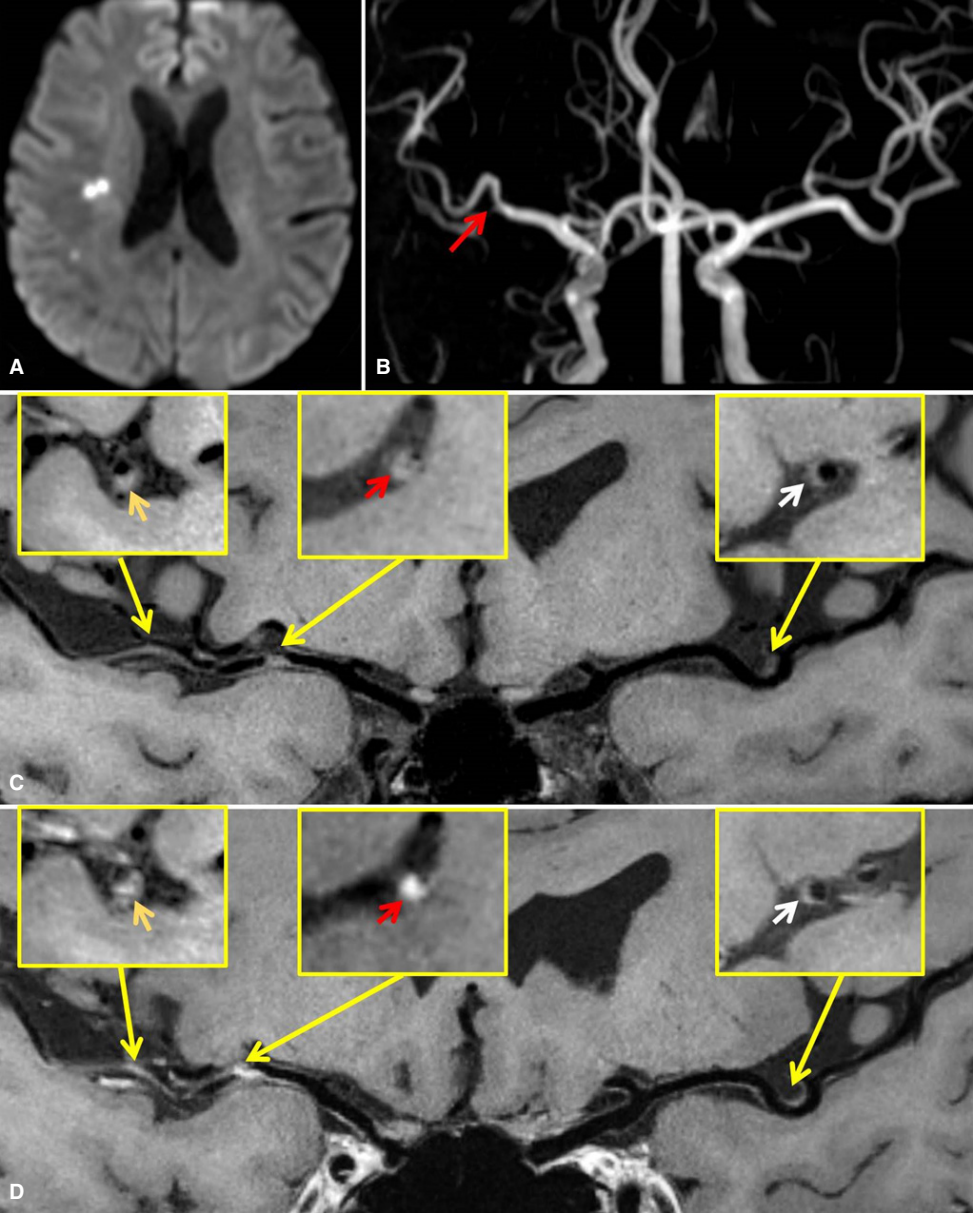
A 62‐year‐old male patient presented with right ischemic stroke. A, Axial diffusion‐weighted imaging detects high signal intensity lesions in the right basal ganglia and corona radiata. B, Time‐of‐flight magnetic resonance angiography image shows a moderate stenosis on the right middle cerebral artery (MCA)M1 segment (red arrow) and no stenosis on the left MCA. C and D, Curved planar reconstruction images from precontrast and postcontrast whole‐brain vessel wall imaging show diffuse and multiple plaques in the right MCA and a focal plaque in the left MCA. Type 2 thickening/enhancement pattern and obvious contrast enhancement (grade 2) are detected in the culprit plaque of the right MCA M1 segment (red arrow). Type 1 thickening/enhancement pattern and mild contrast enhancement (grade 1) are identified in the probable culprit plaque of the right MCA M2 segment (gold arrow) and the nonculprit plaque of the left MCA M1 segment (white arrow).

The incidence of type 2 enhancement pattern was significantly higher (*P*<0.001 for both) in culprit lesions (73.1%) than in probably culprit lesions (21.6%) and nonculprit lesions (10.7%). The enhancement pattern showed no difference between probably culprit lesions and nonculprit lesions (*P*=0.096). A representative case is shown in Figure 3.

### Associations of Plaque Features With Culprit Lesions

**Figure 4:**
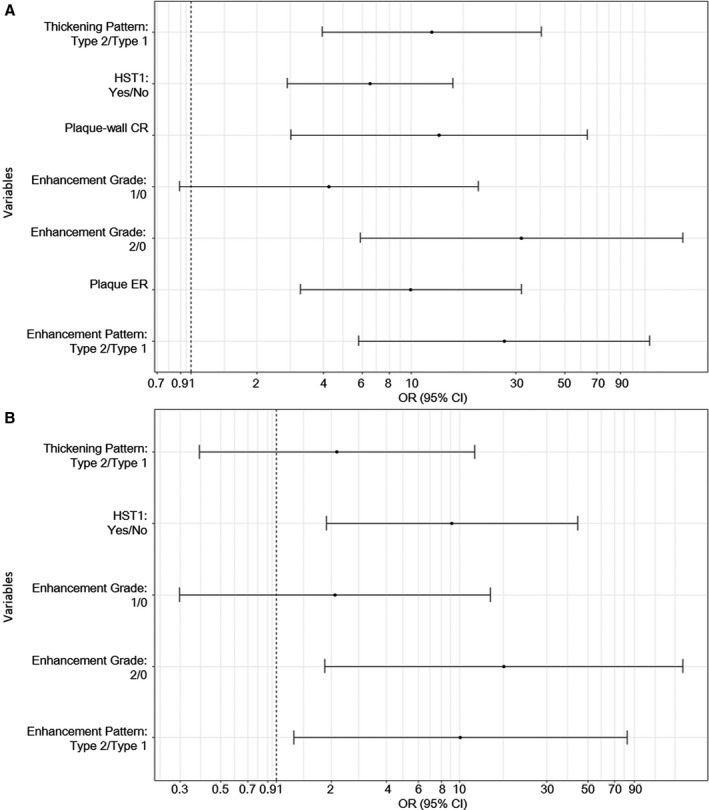
A, Odds ratio (OR) and 95% confidence interval (CI) of culprit lesions vs probably culprit lesions or nonculprit lesions on the basis of a mixed multivariable logistic regression for each feature, separately adjusted by maximum wall thickness. B, OR (95% CI) of culprit lesions vs probably culprit lesions or nonculprit lesions on the basis of a mixed multivariable logistic regression for all features, adjusted by maximum wall thickness. CR indicates contrast ratio; ER, enhancement ratio; HST1, high signal on T1‐weighted images.

After adjustment by maximum wall thickness, type 2 thickening pattern (OR, 12.4; 95% CI, 3.9–39.1; *P*<0.001), HST1 (OR, 6.5; 95% CI, 2.7–15.5; *P*<0.001), plaque‐wall CR (OR, 13.4; 95% CI, 2.8–63.2; *P*=0.001), grade 2 contrast enhancement (OR, 31.9; 95% CI, 5.9–171.9; *P*<0.001), plaque ER (OR, 10.0; 95% CI, 3.1–31.7; *P*<0.001), and type 2 enhancement pattern (OR, 26.6; 95% CI, 5.8–121.9; *P*<0.001) were individually associated with culprit lesions (Figure 4A). According to mixed multivariable logistic regression for all qualitative features adjusted by maximum wall thickness, HST1 (OR, 9.1; 95% CI, 1.9–44.1; *P*=0.006), grade 2 contrast enhancement (OR, 17.4; 95% CI, 1.8–164.9; *P*=0.013), and type 2 enhancement pattern (OR, 10.1; 95% CI, 1.3–82.2; *P*=0.030) were still independently associated with culprit lesions (Figure 4B).

### Reliability of Quantitative Measurements

A strong intraobserver reliability for maximum wall thickness, plaque‐wall CR, and lesion contrast ER was found, with intraclass correlation coefficients (95% CIs) of 0.93 (0.88–0.96), 0.90 (0.84–0.94), and 0.94 (0.90–0.96), respectively. Interobserver reproducibility for the above measurements was also excellent, with intraclass correlation coefficients (95% CIs) of 0.87 (0.80–0.92), 0.89 (0.83–0.94), and 0.90 (0.84–0.94), respectively.

## Discussion

In this study, a group of plaque features from intracranial atherosclerotic lesions affecting the bilateral anterior circulation in patients with acute unilateral ischemic stroke were evaluated. Our study showed that type 2 thickening/enhancement pattern, HST1, and grade 2 contrast enhancement as well as the values of plaque‐wall CR and plaque ER were individually associated with culprit lesions. Furthermore, when considered together and adjusted by maximum wall thickness, HST1, grade 2 contrast enhancement, and type 2 enhancement pattern still demonstrated an independent association with culprit lesions.

In studies of coronary^22^, ^23^ and carotid^24^ atherosclerotic diseases with pathologic confirmation, intraplaque hemorrhage (IPH) is deemed as a good predictor of ischemic events and plaque progression. The degree of vasa vasorum invading from the adventitia is increased in ruptured plaques compared with stable plaques, and microvascular incompetence is a plausible explanation for the occurrence of IPH.^22^, ^25^ Several previous intracranial VWI studies have related HST1 to IPH because of the fact that the major substance of IPH, methemoglobin, has a relatively short T1 relaxation time and, thus, appears hyperintense on T1‐weighted imaging.^13^, ^16^ In their work, HST1 was highly associated with ischemic symptoms.^13^, ^16^ In another study by Natori et al,^18^ HST1 was believed to indicate the presence of lipid components in addition to IPH. Nevertheless, the authors also found that HST1 was more prevalent in the plaques of the middle cerebral artery M1 segment ipsilateral to the acute infarcts. Our study, with a larger cohort, showed that plaques with HST1 were more frequently seen in culprit lesions, supporting the relationship of HST1 and recent stroke.

In the present study, the prevalence of HST1 was 75.0% in culprit lesions. Xu et al^13^ reported HST1 in 19.6% of culprit lesions, and Yu et al^16^ reported HST1 in 53.3% of culprit lesions. Note that our definition of HST1 is based on whether plaque‐wall CR is >150%, whereas previous studies used different criteria. This factor, combined with differential imaging parameter settings for T1‐weighted imaging, needs to be considered when comparing the prevalence of HST1 reported herein with those reported by other studies. A potential reason for the comparatively higher prevalence of HST1 in our work is the substantially improved T1 contrast weighting offered by the WB‐VWI technique.^20^

Studies of carotid atherosclerotic plaques have suggested that contrast enhancement of plaques is a marker of neovascularization and inflammation.^26^, ^27^ Also, a postmortem study found that neovascularity in intracranial atherosclerotic plaque is associated with infarct.^28^ Several studies of intracranial VWI have reported that plaque enhancement is associated with acute, subacute, and even chronic infarction.^7^, ^15^, ^17^, ^29^, ^30^ We found that strong contrast enhancement (grade 2) was associated with culprit lesions, likely suggesting greater neovascularization and/or inflammation activities in those lesions. Similarly, Qiao et al have shown that grade 2 enhancement is associated with culprit plaques.^15^ However, in their work, all the culprit and probably culprit plaques enhanced, and 90% of the culprit plaques showed grade 2 enhancement, demonstrating a higher incidence than our study. This disparity may be attributable to the different patient cohorts. Plaques in the posterior circulation vessels were included in the analysis by Qiao et al^15^ but not in ours. With the development of ICAD, definite and frequent enhancement of the vertebral artery was preferable because of the proliferation of vasa vasorum,^31^, ^32^ which may explain the higher frequency of contrast enhancement in culprit/probably culprit lesions and more obvious enhancement in culprit lesions.

In the present study, type 2 thickening pattern was more prevalent in culprit lesions. This suggests that type 2 thickening pattern may be a marker of more progressive and extensive plaques, which may easily develop into vulnerable plaques. This finding is in line with a previous study, in which the authors found that asymptomatic lesions were associated with eccentric plaques, but symptomatic lesions were both eccentric and concentric.^7^ However, their study included most patients with chronic stroke, which failed to accurately reflect the association between plaque features and acute stroke. Similar results were also found in plaque enhancement patterns in our study. As such, type 2 enhancement pattern may reflect extensive neovascularization or inflammatory cell infiltration, which may, in turn, provide an indication of plaque vulnerability in culprit lesions. Also, an independent association with culprit lesions was observed for type 2 enhancement pattern but not for type 2 thickening pattern when features were considered together. This underscores the value of using MR contrast medium in characterizing plaque vulnerability.

To our best knowledge, this is the first study to explore a group of plaque features in both quantitative and qualitative forms among different types of plaques in a homogeneously acute patient cohort. Although plaque features, such as HST1 and contrast enhancement, may persistent for months of ischemic stroke,^24^, ^33^, ^34^, ^35^ the short period (≤30 days) in our study contributed to the identification of meaningful associations of the features with culprit lesions. However, there is overlap in the studied features among groups. In line with previous intracranial VWI studies, most of the qualitative features are lacking pathologic validation. Thus, to accurately define the characteristics of intracranial culprit lesions, the quantitative features, including CR and ER, were analyzed in our study. The threshold of 1.5 for CR was used to distinguish culprit, probably culprit, and nonculprit lesions. However, this cutoff has been used on the basis of a previous study.^36^ Therefore, the relative importance of HST1 and this CR cutoff compared with other stroke risk factors should be confirmed in a larger prospective study. On the basis of these highly associated features, accurate identification of the culprit lesions in patients with acute stroke may be possible, facilitating clinicians in their treatment toward precise secondary stroke prevention. The findings from this work can also serve as a foundation for future investigations into the imaging markers for high‐risk plaques in patients with asymptomatic ICAD. Early interventions aiming at these high‐risk lesions may effectively reduce the incidence of stroke.

The present study has several limitations. First, we classified all detected plaques into 3 categories on the basis of their stenosis degree and responsible vascular territory, which is current clinical practice because of the lack of a true gold standard. As a result, the assessed plaque features were highly confounded by the lesion's stenosis degree. However, whether the studied features may provide complementary information to stenosis needs to be addressed in longitudinal studies, particularly in equivocal scenarios whereby multiple lesions with similar stenosis degree are responsible for the same vascular territory. Second, the study recruited patients with strokes in the anterior circulation only. Therefore, our conclusion has to be extrapolated to the posterior circulation with caution, given the reported evidence about the morphological difference in plaques between the 2 vascular sites.^8^ Last, the features explored in this study are still limited. A multisequence protocol, although making scan time longer, may help extract additional useful features.

In conclusion, HST1, grade 2 contrast enhancement, and type 2 enhancement pattern are independently associated with culprit lesions. Detailed evaluations of these features in symptomatic or asymptomatic patients may provide valuable insights into risk stratification and, therefore, facilitate preventive therapeutic interventions.

## Sources of Funding

This work was supported in part by the American Heart Association (15SDG25710441), the National Key Research and Development Program of China (2016YFC1301702 and 2017YFC1307903), Beijing Natural Science Foundation (L172043), and the National Science Foundation of China (NSFC 91749127).

## Disclosures

Bi is an employee of Siemens AG Healthcare. The remaining authors have no disclosures to report.
